# Thyroid disorders in obese patients. Does insulin resistance make a difference?

**DOI:** 10.1590/2359-3997000000306

**Published:** 2017-12-01

**Authors:** Nicoleta Răcătăianu, Nicoleta Leach, Cosmina Ioana Bondor, Smaranda Mârza, Daniela Moga, Ana Valea, Cristina Ghervan

**Affiliations:** 1 Iuliu Haţieganu University of Medicine and Pharmacy Department of Endocrinology Cluj Napoca Romania Department of Endocrinology, Iuliu Haţieganu University of Medicine and Pharmacy Cluj-Napoca, Romania; 2 Iuliu Haţieganu University of Medicine and Pharmacy 5^th^ Department of Internal Medicine Cluj Napoca Romania 5^th^ Department of Internal Medicine, Iuliu Haţieganu University of Medicine and Pharmacy Cluj-Napoca, Romania; 3 Iuliu Haţieganu University of Medicine and Pharmacy Department of Medical Informatics and Biostatistics Cluj Napoca Romania Department of Medical Informatics and Biostatistics, Iuliu Haţieganu University of Medicine and Pharmacy Cluj-Napoca, Romania; 4 Infectious Diseases Clinical Hospital-Integrated Ambulatory Pediatrics Cluj Napoca Romania Pediatrics, Infectious Diseases Clinical Hospital-Integrated Ambulatory, Cluj-Napoca, Romania; 5 Infectious Diseases Clinical Hospital-Integrated Ambulatory Laboratory Department Cluj Napoca Romania Laboratory Department, Infectious Diseases Clinical Hospital-Integrated Ambulatory, Cluj-Napoca, Romania

**Keywords:** Insulin resistance, thyroid pathology, metabolic syndrome, obesity

## Abstract

**Objective:**

The aim of this study was to evaluate the association between insulin resistance and thyroid pathology in obese patients, and compare the results between insulin-resistant and noninsulin-resistant patients.

**Subjects and methods:**

Obese/nondiabetic patients, aged 18-70 years, attending the outpatient endocrinology service for 2 years were consecutively included. We evaluated the patients' fasting plasma glucose, insulin, homeostasis model assessment of insulin resistance index (HOMA-IR), thyroid-stimulating hormone (TSH), free thyroxine (FT4), antithyroperoxidase antibodies (TPO-Ab), antithyroglobulin antibodies (Tg-Ab), and thyroid ultrasound.

**Results:**

We included 82 patients with a mean age 44.21 ± 12.67 years. The thyroid disorders encountered and their prevalences were: hypothyroidism (14.6%, 95% confidence interval [CI] 8.6-23.8%), hyperthyroidism (1.2%, 95% CI 2.0-6.6%), goiter (28.0%, 95% CI 19.5-3.6%), thyroid nodules (35.4%, 95% CI 25.9-46.2%), and Hashimoto's thyroiditis (32.9%, 95% CI 23.7-43.7%). HOMA-IR correlated positively with TSH levels (r = 0.24, p = 0.028), and this correlation remained after adjustment for body mass index (BMI), waist/hip ratio (WHR), serum cortisol, subcutaneous fat thickness (SFT), visceral fat thickness (VFT), triglycerides, γ-glutamyl transpeptidase (GGT), and alanine aminotransferase (ALT) in multivariate regression analysis (b = 0.207, 95% CI, 0.09-0.385, p = 0.023). TSH levels were significantly higher in patients with HOMA-IR ≥ 2.5 than in those with HOMA-IR < 2.5 (2.03 μIU/mL, interquartile range [IQR] 1.59-2.69 μIU/mL) versus 1.59 μIU/mL, IQR 0.94-2.26 μIU/mL, p = 0.023).

**Conclusions:**

The most prevalent thyroid disorder in patients attending our endocrinology clinic for investigation of obesity was thyroid nodules. One in seven patients had hypothyroidism. Our findings suggest that TSH levels correlate with insulin resistance in obese patients.

## INTRODUCTION

Thyroid hormones have well-known effects on carbohydrate metabolism, including insulin resistance (IR), which is caused both by hyperthyroidism and hypothyroidism. However, the impact of thyroid hormones on metabolic syndrome (MS) and its main element, IR, in particular, are still unknown. Systemic effects of IR are complex. Among them, the metabolic effects are the most common and include decreased glucose tolerance, type 2 diabetes mellitus, hypercholesterolemia, hypertriglyceridemia, nonalcoholic fatty liver disease (NAFLD), central obesity, hypertension, “low-grade” chronic inflammatory status, oxidative stress, and increased cardiovascular risk.

The endocrine effects of IR are not completely known, although the involvement of IR in the etiopathology of polycystic ovary syndrome (POS) is well established. Lately, there has been increased interest in the effects of IR and MS on the thyroid (1-3). The association of IR and MS with thyroid diseases has been reported by several studies that have shown an increased incidence of hypothyroidism, autoimmune thyroiditis, thyroid nodules, and differentiated thyroid carcinomas along with other systemic malignancies (4,5). Patients with both POS and IR have been described as having an increased frequency of thyroid disorders such as hypothyroidism, Hashimoto's thyroiditis, and Graves’ disease (6). IR may play a role in the etiopathogenesis of thyroid disorders through different mechanisms. Similar to IGF-1, insulin is a growth factor with *in vitro* proliferative, antiapoptotic, angiogenic, and “mitogenic” effects on cultured thyroid cells (7,8). Hyperinsulinemia may increase the volume of the thyroid and lead to the development of thyroid nodules (9,10). Most obese patients with IR present metabolic liver disease as a consequence of disorders in lipid and glucose metabolism induced by hyperinsulinemia, which in turn may affect the hepatic T4 to T3 conversion and the feedback exerted by the free hormone fractions on TSH secretion (5,11). Additionally, IR increases the inflammatory response by stimulating adipocyte production of inflammatory cytokines. Oxidative stress is also increased in MS, and both are involved in the pathogenesis of carcinoma and autoimmune thyroid diseases (3,4). Hypertonia of the hypothalamic-pituitary-adrenal (HPA) axis, which is more pronounced in obesity, may also have an impact on thyroid function (12).

The present study aimed at assessing the relationship between IR and pathologies of the thyroid by performing a morphofunctional thyroid evaluation of a representative sample of obese patients. A secondary objective was to analyze the differences between obese patients with and without IR. We also investigated the association between morning serum cortisol, thyroid function, and IR.

## SUBJECTS AND METHODS

### Patients

This was a cross-sectional, observational study using representative sampling. The cohort comprised all consecutive patients referred for obesity-related issues to the ambulatory of the Department of Endocrinology at the Infectious Diseases Hospital over a period of 2 years.

The inclusion criteria were a body mass index (BMI) > 30 kg/m^2^ and age between 18-70 years. The exclusion criteria comprised a diagnosis of diabetes (history of diabetes or fasting glucose ≥ 125 mg/dL), thyroid disorders, psychiatric disorders, hepatic insufficiency, congestive heart failure, or refusal to participate in the study. All participants underwent complete clinical, laboratory, and ultrasonographic evaluations.

Prior to recruitment, informed consent was obtained from each patient. The study protocol was designed according to the ethical guidelines of the 1975 Declaration of Helsinki and approved by the Ethics Committee at Iuliu Haţieganu University of Medicine and Pharmacy.

### Clinical evaluation

We collected the patients’ personal data, demographic characteristics (gender and age), anthropometric data (weight, height, waist circumference [WC], hip circumference [HC], waist/hip ratio [WHR]), and clinical data (blood pressure).

Body weight (in kilograms) and standing height (in meters) were measured with the patients wearing lightweight clothes and no shoes. BMI (in kg/m^2^) was calculated by dividing each patient's weight (in kilograms) by their squared height (in meters). Obesity was defined as a BMI > 30 kg/m^2^ and subdivided into 1^st^ degree (30-34.9 kg/m^2^), 2^nd^ degree (35-39.9 kg/m^2^), and 3^rd^ degree (≥ 40 kg/m^2^) (13). With the patient standing, WC was measured at the midpoint between the lower border of the rib cage and the iliac crest at the end of expiration, whereas HC was measured at the widest point between the hip and buttocks. The WHR was considered abnormal if > 0.89.

Blood pressure was measured with a mechanical sphygmomanometer. Hypertension or uncontrolled hypertension was defined as the presence of blood pressure values > 140/90 mmHg, according to the Eighth Joint National Committee.

### Laboratory investigation

Venous blood samples were drawn in the morning after overnight fasting. Metabolic parameters (fasting glucose, total cholesterol, HDL-cholesterol, LDL- cholesterol, aspartate aminotransferase [AST], alanine aminotransferase [ALT], γ-glutamyl transpeptidase [GGT], and uric acid) were assayed on an automatic analyzer (Beckman Coulter Unicell DXC600, Beckman Coulter Inc., Fullerton, CA, USA) using standard laboratory procedures. Immunological parameters were analyzed with the same automatic analyzer (Beckman Coulter Unicell DXI 600) by ELISA method, following specifications of the kit's protocol. Serum insulin was measured, and the degree of IR was calculated according to the homeostasis model assessment of IR (HOMA-IR) using the formula: (fasting plasma glucose [mg/dL] x fasting serum insulin [μU/mL])/405. A HOMA-IR cutoff value of 2.5 defined IR (14,15). MS was defined according to the 2009 Joint Interim Statement of the International Diabetes Federation Task Force on Epidemiology and Prevention, National Heart, Lung, and Blood Institute, American Heart Association, World Heart Federation, International Atherosclerosis Society and International Association for the Study of Obesity. According to this definition, the patients were classified as having MS if at least three of the following five criteria were present: 1) WC > 94 cm in men or > 80 cm in women, 2) triglycerides > 150 mg/dL or fibrate treatment, 3) HDL-cholesterol < 40 mg/dL in men and < 50 mg/dL in women or cholesterol-lowering treatment, 4) fasting plasma glucose > 100 mg/dL or previously diagnosed type 2 diabetes mellitus, 5) blood pressure > 130/85 mmHg or antihypertensive treatment (16).

Thyroid function was assessed with measurement of TSH and free T4 serum levels, while screening for Hashimoto's thyroiditis was done with determination of antithyroid peroxidase (TPO) and antithyroglobulin antibodies. Serum cortisol was measured at 8 a.m. in all patients to assess the HPA axis and evaluate the relationship between serum cortisol levels and BMI, WC, WHR, serum insulin, HOMA-IR, and thyroid function.

### Ultrasound evaluation

Each participant was evaluated with thyroid ultrasound for assessment of thyroid volume and morphology and detection of goiter, nodules, and autoimmune thyroiditis. The ultrasound evaluations were performed on a DC-N3 Doppler ultrasound system (Mindray DS USA, Inc., Mahwah, NJ, USA) equipped with a 7.5 MHz linear transducer. We also performed abdominal ultrasound evaluations to detect the occurrence of hepatic steatosis and measured the subcutaneous fat thickness (SFT) and visceral fat thickness (VFT) using the same ultrasound equipment (Mindray DC-N3 Doppler) equipped with a 5 MHz convex transducer. Hepatic steatosis was defined as the presence of a diffuse, hyperechoic, and bright liver, with an increased echotexture when compared to the kidneys, vascular blurring, and deep attenuation of the ultrasonic beam. The occurrence of nonalcoholic steatohepatitis (NASH) was defined by the presence of a combination of imaging (hepatic steatosis on ultrasound imaging) and laboratory criteria (detection of increased transaminases levels after exclusion of other causes of secondary hepatic fat accumulation, such as significant alcohol consumption [> 30 g alcohol/day for men and > 20 g/day for women], other liver diseases [hepatitis B, C, and autoimmune hepatitis], use of steatogenic medications, or hereditary disorders) (17).

The VFT was measured with the ultrasound probe located 1 cm above the umbilicus on the xiphoumbilical line in both longitudinal and transverse views and defined as the distance between the *linea alba* and the anterior wall of the aorta. The SFT was determined as the distance between the *linea alba* and the skin, with the transducer located 1 cm above the umbilicus. All patients were evaluated by the same radiologist.

### Statistical analysis

All results were statistically analyzed using Excel (Microsoft, Redmond, WA, USA) and SPSS (SPSS, Inc., Chicago, IL, USA). The sample size was calculated based on a 0.1-0.3% prevalence of thyroid pathologies with a 95% level of confidence and ± 10% confidence interval (CI) length (3,18). Data with normal distribution were reported as mean ± standard deviation, and those without normal distribution as median (25^th^ – 75^th^ percentiles [Q1, Q3]). Differences between groups were analyzed with Student's *t* test for independent samples in case of normally distributed quantitative variables, and the Mann-Whitney test for data without normal distribution. For qualitative data, chi-square test of Fisher's exact test was used. Pearson's correlation coefficient was applied to determine the linear relationship between two or more continuous quantitative variables, while Spearman's correlation coefficient was used to assess the relationship between several continuous quantitative variables with outliers or to assess nonlinear relationships.

In order to estimate the relationship between serum TSH levels and HOMA-IR values with multivariate analysis, we used multiple logistic regression. We adopted as the dependent variable the classification given by the HOMA-IR cutoff value and as independent variables all other continuous variables that correlated significantly with HOMA-IR in the univariate analysis. Considering that the relationship between HOMA-IR values and serum TSH levels was nonlinear, we adopted the classification given by the ratio between HOMA-IR values and TSH levels. Using a HOMA-IR cutoff value of 2.5, the patients were divided into two groups: obese and IR (HOMA-IR ≥ 2.5; Ob-IR) and obese without IR (HOMA-IR < 2.5; Ob-NIR).

## RESULTS

### Baseline characteristics

The study included 82 obese patients with a mean age of 44.21 ± 12.67 years, of whom 91.5% were women, and 8.5% were men. Table 1 describes the clinical, laboratory, and ultrasonographic characteristics of the cohort. Most patients (n = 48; 58.5%) had a BMI between 30 and 35 kg/m^2^. Overall, 22 (26.8%) had a BMI between 35 and 40 kg/m^2^, while 12 (14.6%) had severe obesity with a BMI above 40 kg/m^2^. A total of 57.3% of the patients met the criteria for MS.

**Table 1:** Clinical and laboratory characteristics of the investigated group

Parameters	Total (n = 82)
Male gender (number; %)	7 (8.5)
Age (years)	44.21 ± 12.67
Weight (kg)	91.5 (85, 103)
Height (cm)	162 (157, 166)
BMI (kg/m^2^)	34.3 (32.38, 37.88)
WC (cm)	108 (102, 116)
HC (cm)	111 (108, 119)
WHR (cm)	0.97 (0.92, 1.03)
HBP (number; %)	39 (47.6)
SBP (mmHg)	130 (120, 140)
DBP (mmHg)	80 (70, 90)
Insulin (μUI/mL)	12.48 ± 5
FPG (mg/dL)	95 (90, 103)
HOMA-IR	2.68 (1.98, 3.9)
TSH (μIU/mL)	1.89 (1.2, 2.66)
FT4 (ng/dL)	0.78 (0.73, 0.88)
TPO-Ab (UI/mL)	1.3 (0.5, 10.6)
Tg-Ab (UI/mL)	0.4 (0.2, 0.9)
Thyroid volume (mL)	12.3 (10.2, 13.7)
Serum cortisol at 8 a.m. (μg/dL)	11.06 (9.05, 14.8)
TG (mg/dL)	133 (104, 166)
LDL-cholesterol (mg/dL)	124.15 ± 28.89
Total cholesterol (mg/dL)	202.16 ± 39.15
HDL-cholesterol (mg/dL)	45.7 ± 9.95
Uric acid (mg/dL)	4.93 ± 1.13
ALT (U/L)	22 (18, 29)
AST (U/T)	20.5 (18, 25)
GGT (U/L)	19 (15, 28)
Hepatic steatosis (number; %)	61 (75.3)
NASH (number; %)	3 (3.7)
MS (number; %)	47 (57.3)
SFT (cm)	2.74 (2.52, 3.09)
VFT (cm)	4.99 ± 1.01

BMI: body mass index; WC: waist circumference; HC: hip circumference; WHR: waist/hip ratio; HBP: high blood pressure; SBP: systolic blood pressure; DBP: diastolic blood pressure; FPG: fasting plasma glucose; HOMA-IR: homeostasis model assessment of insulin resistance index; TSH: thyroid-stimulating hormone; FT4: free thyroxine; TPO-Ab: antithyroid peroxidase antibodies; Tg-Ab: antithyroglobulin antibodies; TG- triglycerides; LDL-cholesterol: low-density lipoprotein cholesterol; HDL-cholesterol: high-density lipoprotein cholesterol; ALT: alanine aminotransferase; AST: aspartate aminotransferase; GGT-γ: gamma glutamyl transpeptidase; NASH: nonalcoholic steatohepatitis; MS: metabolic syndrome; SFT: subcutaneous fat thickness; VFT: visceral fat thickness. The results are expressed as mean ± standard deviation (SD) or median and range (25-75%).

Table 2 shows the frequency of thyroid disorders in the cohort. Thyroid nodules were among the most frequent morphological abnormalities, while one out of seven patients presented hypothyroidism.

**Table 2:** Frequency of thyroid disorders in the overall study cohort

Parameters	Total (n = 82)	95% confidence interval (percentages)
Hypothyroidism (number; %)	12 (14.6)	8.6-23.8
Euthyroid (number; %)	69 (84.1)	74.8-90.5
Hyperthyroidism (number; %)	1 (1.2)	0.2-6.6
Goiter (number; %)	23 (28.0)	19.5-38.6
Thyroid nodules (number; %)	29 (35.4)	25.9-46.2
Hashimoto's thyroiditis (number; %)	27 (32.9)	23.7-43.7

### Analysis of the relationship between HOMA-IR and thyroid function

There was a positive correlation between HOMA-IR values and TSH levels (r = 0.24, p = 0.028) in the overall cohort (n = 82). TSH correlated positively with serum insulin (r = 0.23, p = 0.037). In contrast, no significant correlation was found between insulin, HOMA-IR, and thyroid volume.

Table 3 presents an analysis of differences between the groups with HOMA-IR ≥ 2.5 and < 2.5 (Ob-IR and Ob- NIR, respectively). Patients with HOMA-IR ≥ 2.5 had significantly higher TSH levels than those with HOMA- IR < 2.5. Thyroid nodules were detected in 44.4% of the patients with HOMA-IR ≥ 2.5 compared with 24.3% of those with HOMA-IR < 2.5 (p = 0.058), thus showing a trend towards an association between both variables. Although patients with HOMA-IR ≥ 2.5 had an increased frequency of goiter and Hashimoto's thyroiditis, the difference was not significant (Table 3). No correlation was found between HOMA-IR values and FT4 levels.

**Table 3:** Comparison of thyroid parameters between the groups with and without insulin resistance (HOMA-IR ≥ 2.5 and < 2.5, respectively)

	HOMA-IR < 2.5 (n = 37)	HOMA-IR ≥ 2.5 (n = 45)	p
Thyroid disorders			0.184
Hypothyroidism (number; %)	3 (8.1)	9 (20.0)	
Euthyroidism (number; %)	33 (89.2)	36 (80.0)	
Hyperthyroidism (number; %)	1 (2.7)	0 (0.0)	
Goiter (number; %)	8 (21.6)	15 (33.3)	0.240
Thyroid nodules (number; %)	9 (24.3)	20 (44.4)	**0.058**
Hashimoto's thyroiditis (number; %)	12 (32.4)	15 (33.3)	0.931
TSH (μIU/mL) (median (Q1, Q3))	1.59 (0.94, 2.26)	2.03 (1.59, 2.69)	**0.023**
FT4 (ng/dL) (median (Q1, Q3))	0.79 (0.72, 0.88)	0.78 (0.73, 0.88)	0.860
TPO-Ab (UI/mL) (median (Q1, Q3))	1.4 (0.5, 10.1)	1.1 (0.4, 10.6)	0.593
Tg-Ab (UI/mL) (median (Q1, Q3))	0.6 (0.2,1.1)	0.4 (0.2, 0.5)	0.226
Thyroid volume (mL) (median (Q1, Q3))	12 (10.2, 13.7)	12.3 (10.3, 13.7)	0.755

HOMA-IR: homeostasis model assessment of insulin resistance index; TSH: thyroid-stimulating hormone; FT4: free thyroxine; TPO-Ab: antithyroid peroxidase antibodies; Tg-Ab: antithyroglobulin antibodies.

### Analysis of the relationship between HOMA-IR and metabolic parameters

As expected, HOMA-IR correlated positively with the degree of obesity, BMI (r = 0.57, p < 0.001), central adipose tissue (WC; r = 0.48, p = 0.000), WHR (r = 0.30, p = 0.006), VFT (r = 0.35, p = 0.002), and SFT (r = 0.28, p = 0.012). HOMA-IR also correlated positively with triglycerides (r = 0.25, p = 0.026), ALT (r = 0.37, p = 0.001), and GGT (r = 0.32, p = 0.003) levels, as well as with 8 a.m. cortisol levels (r = 0.24, p = 0.03).

Both groups also presented differences in metabolic parameters (BMI, WC, HC, SFT, VFT) and MS, which were significantly higher in patients with HOMA-IR ≥ 2.5 than in those with HOMA-IR < 2.5 (Table 4).

**Table 4:** Comparison of metabolic parameters between the groups with and without insulin resistance (HOMA -IR ≥ 2.5 and < 2.5, respectively)

	HOMA-IR < 2.5 (n = 37)	HOMA-IR ≥ 2.5 (n = 45)	p
Male gender, n (%)	4 (10.8)	3 (6.7)	0.695
Age (years), mean ± SD	44.92 ± 12.64	43.62 ± 12.82	0.648
Hepatic steatosis, n (%)	25 (67.6)	36 (81.8)	0.138
NASH, n (%)	1 (2.7)	2 (4.4)	1.00
Uncontrolled HBP (mmHg), n (%)	12 (32.4)	25 (55.6)	**0.046**
MS, n (%)	15 (40.5)	32 (71.1)	**0.005**
BMI (kg/m^2^), median (Q1, Q3)	33.1 (32, 36)	35.8 (33.8, 39.4)	**0.000**
SBP (mmHg), median (Q1, Q3)	120 (120, 130)	140 (120, 145)	0.227
DBP (mmHg), median (Q1, Q3)	80 (70, 90)	85 (70, 90)	0.719
WC (cm), median (Q1, Q3)	102 (98, 113)	112 (106, 116)	**0.003**
HC (cm), median (Q1, Q3)	110 (107, 117)	113 (110, 121)	**0.017**
WHR (cm), median (Q1, Q3)	0.95 (0.91,1.02)	0.982 (0.922, 1.04)	0.124
Insulin (μUI/mL), mean ± SD	7.8 ± 1.9	16.33 ± 5.13	**0.000**
FPG (mg/dL), median (Q1, Q3)	93 (89, 99)	99 (93, 106)	**0.009**
HOMA-IR, median (Q1, Q3)	1.95 (1.5, 2.1)	3.59 (3.08, 4.43)	
TG (mg/dL), median (Q1, Q3)	126 (92, 166)	139 (121, 165)	0.252
LDL-cholesterol (mg/dL), mean ± SD	122.16 ± 25.2	125.78 ± 31.79	0.576
Total cholesterol(mg/dL), mean ± SD	198.86 ± 36.01	204.87 ± 41.77	0.493
HDL-cholesterol (mg/dL), mean ± SD	46.28 ± 9.96	45.22 ± 10.02	0.636
Uric acid (mg/dL), mean ± SD	4.84 ± 1.08	5 ± 1.18	0.504
ALT (U/L), median (Q1, Q3)	19 (17, 25)	24 (19, 36)	0.089
AST (U/T), median (Q1, Q3)	20 (19, 23)	21 (18, 26)	0.690
GGT (U/L), median (Q1, Q3)	17 (14, 25)	19 (16, 29)	0.138
Serum cortisol (μg/dL), median (Q1, Q3)	11.11 (8.38, 14.6)	11.01 (9.63, 15.78)	0.134
SFT (cm), median (Q1, Q3)	2.62 (2.41,2.86)	2.90 (2.66, 3.3)	**0.000**
VFT (cm), mean ± SD	4.74 ± 0.92	5.19 ± 1.05	**0.052**

NASH: nonalcoholic steatohepatitis; HBP: high blood pressure; MS: metabolic syndrome; BMI: body mass index; SBP: systolic blood pressure; DBP: diastolic blood pressure; WC: waist circumference; HC: hip circumference; WHR: waist/hip ratio; FPG: fasting plasma glucose; HOMA-IR: homeostasis model assessment of insulin resistance index; TG: triglycerides; LDL-cholesterol: low-density lipoprotein cholesterol; HDL-cholesterol: high-density lipoprotein cholesterol; ALT: alanine aminotransferase; AST: aspartate aminotransferase; GGT-γ: gamma glutamyl transpeptidase; SFT: subcutaneous fat thickness; VFT: visceral fat thickness. The results are expressed as mean ± standard deviation (SD) or median and range (25-75%).

### Analysis of the relationship between serum TSH levels and metabolic parameters

Serum TSH levels correlated positively with serum triglycerides levels (r = 0.30, p = 0.006) and negatively with serum HDL-cholesterol levels (r = −0.25, p = 002). There was a positive relationship between serum TSH levels and serum morning cortisol levels (r = 0.26, p = 0.02).

When we compared obese patients with and without hepatic steatosis in regards to thyroid dysfunction, we found no significant differences in TSH levels between both groups. Obese patients with NASH displayed significantly higher TSH values compared with those without NASH (Table 5). There were no significant differences in TSH levels between subjects with versus those without MS.

**Table 5:** TSH levels (expressed as median and (Q1, Q3)) according to the presence or not of hepatic steatosis, nonalcoholic steatohepatitis, and metabolic syndrome

	Without MS (n = 35)	With MS (n = 47)	p
TSH	1.75 (1.10, 2.53)	1.99 (1.34, 2.68)	0.36
	Without NASH (n = 79)	With NASH (n = 3)	p
TSH	1.77 (1.19, 2.49)	4.76 (3.73, 4.80)	0.04
	Without hepatic steatosis (n = 20)	With hepatic steatosis (n = 61)	p
TSH	1.61 (1.02, 2.42)	1.99 (1.29, 2.66)	0.21

TSH: thyroid-stimulating hormone; NASH: nonalcoholic steatohepatitis; MS: metabolic syndrome.

### Multivariate analyses

On multivariate linear analysis including the model with HOMA-IR cutoff values as a dependent variable and all other quantitative variables as independent variables, the correlation between serum TSH levels and HOMA-IR values remained (b = 0.195, 95% CI 0.0140.376, p = 0.035). The covariate variables were BMI, WHR, serum cortisol, SFT, VFT, triglycerides, GGT, and ALT. Serum cortisol also predicted statistically significant HOMA-IR (b = 1.535, 95% CI 0.2822.789, p = 0.017) along with BMI and TSH.

## DISCUSSION

The results of this study showed a significant positive correlation of HOMA-IR values with serum TSH levels among obese patients. Ob-IR patients had significantly higher TSH levels compared with Ob-NIR patients, although FT4 levels were comparable in both groups (Table 3). These results are consistent with others reported in the literature (19,20). Increased TSH level could thus be hypothesized to be a metabolic consequence of obesity. The mechanisms by which IR may impact the thyroid are not completely understood. Recent evidence suggests that the main suspected mechanism is a possible relationship between thyroid hormones and the adipokines released by visceral fat, especially leptin (21,22). Leptin interferes with the negative feedback regulation of thyroid hormones and stimulates the hypothalamic-pituitary-thyroid axis to increase TSH secretion (23). Production of leptin increases along with increased body fat, with visceral fat mass inducing and aggravating IR, while hyperinsulinemia stimulates the production of leptin (24).

Another hypothesis is that the metabolic hepatic disorder related to IR could impact the metabolism of thyroid hormones. Hepatic IR leads to increased glucose production and increased VLDL-cholesterol, aggravating hyperglycemia and hyperinsulinemia. IR increases the influx of free fatty acids (FFA) to the liver and *de novo* lipogenesis, leading to lipid accumulation in hepatocytes, which in the context of the inflammatory status and oxidative stress, increases lipid peroxidation and mitochondrial dysfunction, leading to NASH (25). Consequently, metabolic liver damage can lead to impaired synthesis of thyroid hormones transport proteins (thyroxine-binding globulin [TBG], albumin, and transthyretin), decreasing the transport of these hormones to target cells. Moreover, decreased type I 5’-deiodinase activity alters T4 to T3 conversion, which can stimulate TSH production by reducing the feedback of the thyroid hormones on the HPA (11,26). In support of the previous hypothesis, HOMA-IR correlated positively with liver markers (ALT and GGT) and obese patients with severe hepatic impairment (NASH) had significantly higher TSH levels than those without NASH (Table 5). Of note, we did not perform liver biopsy for histological confirmation of NASH, which may be considered one of our study's limitations.

As expected, HOMA-IR correlated positively with the degree of obesity (BMI) and visceral adipose tissue (WC, WHR, and VFT) and the Ob-IR group had significantly higher BMI, VFT, and WC values when compared with the Ob-NIR group (Table 4). This fact supports the theory that obesity and particularly its visceral component should be considered the main causative factor in MS (27). Macrophage infiltration with increased production of adipokines and cytokines (TNF-α, MCP-1, CRP, IL-1, IL-6, FFA, leptin, resistin) occurs at the level of visceral fat, leading to chronic “low-grade” inflammation and oxidative stress, which affect insulin signaling in target cells (and binding of insulin to its receptor) leading to IR and its systemic consequences (28).

A comparison of both our study groups showed a significantly higher incidence of MS and uncontrolled hypertension among Ob-IR patients. Also, the OB-IR group had a higher frequency of liver disease (NAFLD/ NASH) than the Ob-NIR group (Table 4).

Recent publications have suggested the occurrence of peripheral resistance to thyroid hormones secondary to the oxidative stress and inflammatory status related to IR, which would alter the transmembrane intracellular transport of thyroid hormone and its binding to nuclear receptors (29).

Other possible assumptions are that the central hypertonia of the TRH-TSH axis in obesity is an adaptation to chronically increased caloric intake aiming at resetting the basal metabolism at a higher level. This results in increased T4 levels and T4 to T3 conversion by stimulation of the type 2 deiodinase (5’D2) activity in muscle and brown tissue to boost energy expenditure. This theory might also justify the higher TSH levels observed among obese patients (26).

Our study has shown a positive correlation between serum TSH and cortisol levels, both of which correlated positively with HOMA-IR. No correlation was found between cortisol and FT4 levels, which is consistent with previously reported data in the specialized literature (30).

Little is known about the HPA axis hypertonia involved in obesity and its relation to the TRH-TSH- thyroid axis. Elevated serum cortisol may have peripheral and hepatic effects by decreasing the hepatic synthesis of TBG, affecting T4 to T3 conversion (decreasing the type 1 deiodinase [5’D1] activity), and stimulating the conversion of T4 into inactive reverse T3 (increasing the type 3 deiodinase [5’D3] activity). Elevated cortisol may also have a central impact since cortisol has an effect on TSH depression (31,32). Moreover, the serum cortisol measurements in our obese patients demonstrated a U-shaped association with BMI: patients with overweight and first-degree obesity showed normal or low morning serum cortisol, which increased along with the obesity (33). These controversies are likely due to the increased peripheral metabolism of cortisol. Patients with obesity may display enhanced inactivation of cortisol by 5α-reductase or impaired reactivation of cortisol from cortisone by 11β-hydroxysteroid dehydrogenase type 1, resulting in activation of the HPA axis (34,35).

A negative correlation between serum TSH and cortisol levels has been reported for TSH cutoff values < 2.0 μUI/mL, while a positive correlation has been observed for cutoff values > 2.0 μUI/mL (30). Similar to our results, other researchers have also reported a positive correlation of cortisol and TSH levels in euthyroid obese patients (36).

Recent studies have shown that MS and its central factor (IR) are associated with increased thyroid volume, prevalence of thyroid nodules, and risk of systemic malignancies, including differentiated thyroid carcinomas (7,37).

Comparing both our groups with and without IR in regards to thyroid morphology, we observed a higher frequency of goiter (33.3% versus 21.6%) and thyroid nodules (44.4% versus 24.3%, p = 0.058) in the Ob- IR group, although without statistical significance. There were no significant differences in the incidence of Hashimoto's thyroiditis in the two groups.

We must note that the results obtained in our study derived from a population attending an Endocrinology Department for investigation of obesity, which explains the 10-fold higher prevalence of women over men in our cohort. As a consequence, the prevalence rates encountered cannot be generalized to the entire population of obese individuals. The female population is known to be the most interested group in investigating the causes of obesity, and multiple studies have been conducted exclusively in women (38). Additionally, thyroid disorders are considered to be more common in women (39). Due to limited financial resources, we calculated the sample size with an accurate CI of ± 10%. We consider this as a limitation of the study and recommend that further research should be conducted including a larger sample size.

The proliferative effect of insulin has been shown *in vitro* using cultured thyroid cells via the insulin receptors and IGF-1 receptor - both overexpressed in thyroid tumors as well as in non-thyroid tumors (breast, colon, liver). Hyperinsulinemia can, thus, determine an increase in thyroid volume, occurrence of thyroid nodules, and thyroid carcinogenesis (4,8,40). Moreover, TSH is a major growth factor in the thyroid gland and a regulator of the expression other growth factors; TSH promotes the insulin/IGF-1 signaling pathway, while IGF-1 is actively involved in TSH- mediated proliferation of thyrocytes (8,41). Patients with IR have higher TSH levels than those without IR and, as a consequence, a higher risk of proliferation of thyroid cells due to its morphogenic effect.

In conclusion, obese patients displayed a significantly positive correlation between HOMA-IR values and TSH levels. Our findings validate the cutoff value of 2.5 for HOMA-IR in regards to TSH levels, which was higher in obese patients with IR when compared with those without IR. Although serum cortisol correlated with HOMA-IR values and serum TSH levels, the correlation of HOMA-IR and TSH remained even after adjusting the cortisol's influence.

## References

[1] Ayturk S, Gursoy A, Kut A, Anil C, Nar A, Tutuncu NB. Metabolic syndrome and its components are associated with increased thyroid volume and nodule prevalence in a mild-to-moderate iodine-deficient area. Eur J Endocrinol. 2009;161(4):599-605. 10.1530/EJE-09-0410 19633072

[2] Topsakal S, Yerlikaya E, Akin F, Kaptanoglu B, Erürker T Relation with HOMA-IR and thyroid hormones in obese Turkish women with metabolic syndrome. Eat Weight Disord. 2012;17(1):e57-61. 10.1007/BF03325329 22751273

[3] Sousa PA, Vaisman M, Carneiro JR, Guimarães L, Freitas H, Pinheiro MF, et al. Prevalence of goiter and thyroid nodular disease in patients with class III obesity. Arq Bras Endocrinol Metabol. 2013;57(2):120-5. 10.1590/s0004-27302013000200004 23525289

[4] Rezzonico JN, Rezzonico M, Pusiol E, Pitoia F, Niepomniszcze H. Increased prevalence of insulin resistance in patients with differentiated thyroid carcinoma. Metab Syndr Relat Disord. 2009;7(4):375-80. 10.1089/met.2008.0062 19320560

[5] Gyawali P, Takanche JS, Shrestha RK, Bhattarai P, Khanal K, Risal P, et al. Pattern of thyroid dysfunction in patients with metabolic syndrome and its relationship with components of metabolic syndrome. Diabetes Metab J. 2015;39(1):66-73. 10.4093/dmj.2015.39.1.66 PMC4342539 25729715

[6] Anaforoglu I, Topbas M, Algun E. Relative associations of polycystic ovarian syndrome vs metabolic syndrome with thyroid function, volume, nodularity and autoimmunity. J Endocrinol Invest. 2011;34(9):e259-64. 10.3275/7681 21521934

[7] Duran AO, Anil C, Gursoy A, Nar A, Altundag O, Tutuncu NB. The relationship between glucose metabolism disorders and malignant thyroid disease. Int J Clin Oncol. 2013;18(4):585-9. 10.1007/s10147-012-0435-3 22752254

[8] Vella V, Sciacca L, Pandini G, Mineo R, Squatrito S, Vigneri R, et al. The IGF system in thyroid cancer: new concepts. Mol Pathol. 2001;54(3):121-4. 10.1136/mp.54.3.121 PMC1187048 11376121

[9] Studer H, Derwahl M. Mechanisms of nonneoplastic endocrine hyperplasia--a changing concept: a review focused on the thyroid gland. Endocr Rev. 1995;16(4):411-26. 10.1210/edrv-16-4-411 8521787

[10] Pothiwala P, Jain SK, Yaturu S. Metabolic syndrome and cancer. Metab Syndr Relat Disord. 2009;7(4):279-88. 10.1089/met.2008.0065 PMC3191378 19284314

[11] Pacifico L, Bonci E, Ferraro F, Andreoli G, Bascetta S, Chiesa C. Hepatic steatosis and thyroid function tests in overweight and obese children. Int J Endocrinol. 2013;2013:381014. 10.1155/2013/381014 PMC3575668 23431294

[12] Nadolnik L. Role of Glucocorticoids in Regulation of Iodine Metabolism in Thyroid Gland: Effects of Hyper- and Hypocorticism. “Glucocorticoids - New Recognition of Our Familiar Friend” - Xiaoxiao Qian, Chapter 12, Published: Nov. 28, 2012. Available from: 10.5772/52043.

[13] WHO. Obesity: preventing and managing the global epidemic. Report of a WHO Consultation. WHO Technical Report Series 894. Geneva 2011: World Health Organization (accessed in May 2013). Available from: http://whqlibdoc.who.int/trs/WHOTRS_894. 11234459

[14] Beineke M. Marker for the diagnosis of insulin resistance. Labor Bioscientia. Available from: www.bioscientia.de.RefType: Internet Communication.

[15] Matthews DR, Hosker JP, Rudenski AS, Naylor BA, Treacher DF, Turner RC. Homeostasis model assessment: insulin resistance and beta-cell function from fasting plasma glucose and insulin concentrations in man. Diabetologia. 1985;28(7):412-9. 10.1007/BF00280883 3899825

[16] Alberti KG, Eckel RH, Grundy SM, Zimmet PZ, Cleeman JI, Donato KA, et al. Harmonizing the metabolic syndrome: a joint interim statement of the International Diabetes Federation Task Force on Epidemiology and Prevention; National Heart, Lung, and Blood Institute; American Heart Association; World Heart Federation; International Atherosclerosis Society; and International Association for the Study of Obesity. Circulation 2009;120(16):1640-45. 10.1161/CIRCULATIONAHA.109.192644 19805654

[17] Chalasani N, Younossi Z, Lavine JE, Diehl AM, Brunt EM, Cusi K, et al. The diagnosis and management of non-alcoholic fatty liver disease: practice Guideline by the American Association for the Study of Liver Diseases, American College of Gastroenterology, and the American Gastroenterological Association. Hepatology. 2012;55(6):2005-23. 10.1002/hep.25762 22488764

[18] Dauksiene D, Petkeviciene J, Klumbiene J, Verkauskiene R, Vainikonyte-Kristapone J, Seibokaite A, et al. Factors Associated with the Prevalence of Thyroid Nodules and Goiter in Middle- Aged Euthyroid Subjects. Int J Endocrinol. 2017;2017:8401518. 10.1155/2017/8401518 PMC5357546 28356911

[19] De Pergola G, Ciampolillo A, Paolotti S, Trerotoli P, Giorgino R. Free triiodothyronine and thyroid-stimulating hormone are directly associated with waist circumference, independently of insulin resistance, metabolic parameters and blood pressure in overweight and obese women. Clin Endocrinol (Oxf). 2007;67(2):265-9. 10.1111/j.1365-2265.2007.02874.x 17547687

[20] Rotondi M, Leporati P, La Manna A, Pirali B, Mondello T, Fonte R, et al. Raised serum TSH levels in patients with morbid obesity: is it enough to diagnose subclinical hypothyroidism? Eur J Endocrinol. 2009;160(3):403-8. 10.1530/EJE-08-0734 19073832

[21] Menendez C, Baldelli R, Camiña JP, Escudero B, Peino R, Dieguez C, et al. TSH stimulates leptin secretion by a direct effect on adipocytes. J Endocrinol. 2003;176(1):7-12. 10.1677/joe.0.1760007 12525244

[22] Feldt-Rasmussen U. Thyroid and leptin. Thyroid. 2007;17:413-9. 10.1089/thy.2007.0032 17542671

[23] Sari R, Balci MK, Altunbas H, Karayalcin U. The effect of body weight and weight loss on thyroid volume and function in obese women. Clin Endocrinol (Oxf). 2003;59(2):258-62. 10.1046/j.1365-2265.2003.01836.x 12864805

[24] Zimmermann-Belsing T, Brabant G, Holst JJ, Feldt-Rasmussen U. Circulating leptin and thyroid dysfunction. Eur J Endocrinol. 2003;149(4):257-71. 10.1530/eje.0.1490257 14514340

[25] Day CP, James OF. Steatohepatitis: a tale of two “hits”? Gastroenterology. 1998;114(4):842-5. 10.1016/s0016-5085(98)70599-2 9547102

[26] Farasat T, Cheema MA, Khan MN. Hyperinsulinemia and insulin resistance is associated with low T3/T4 ratio in prediabetic euthyroid Pakistani subjects. J Diabetes Complications. 2012;26(6):522-5. 10.1016/j.jdiacomp.2012.05.017 22795338

[27] Freedland ES. Role of a critical visceral adipose tissue threshold (CVATT) in metabolic syndrome: implications for controlling dietary carbohydrates: a review. Nutr Metab (Lond). 2004;1(1):12. 10.1186/1743-7075-1-12 PMC535537 15530168

[28] Weisberg SP, McCann D, Desai M, Rosenbaum M, Leibel RL, Ferrante AW Jr. Obesity is associated with macrophage accumulation in adipose tissue. J Clin Invest. 2003;112(12):1796-808. 10.1172/JCI19246 PMC296995 14679176

[29] Holtorf K. Thyroid Hormone Transport into Cellular Tissue. J Restorative Medicine. 2014;3(1):53-68.

[30] Walter KN, Corwin EJ, Ulbrecht J, Demers LM, Bennett JM, Whetzel CA, et al. Elevated thyroid stimulating hormone is associated with elevated cortisol in healthy young men and women. Thyroid Res. 2012;5(1):13. 10.1186/1756-6614-5-13 PMC3520819 23111240

[31] Samuels MH, McDaniel PA. Thyrotropin levels during hydrocortisone infusions that mimic fasting-induced cortisol elevations: a clinical research center study. J Clin Endocrinol Metab. 1997;82(11):3700-4. 10.1210/jcem.82.11.4376 9360528

[32] Kokkoris P, Pi-Sunyer FX. Obesity and endocrine disease. Endocrinol Metab Clin North Am. 2003;32(4):895-914. 10.1016/s0889-8529(03)00078-1 14711067

[33] Schorr M, Lawson EA, Dichtel LE, Klibanski A, Miller KK. Cortisol Measures Across the Weight Spectrum. J Clin Endocrinol Metab. 2015;100(9):3313-21. 10.1210/JC.2015-2078 PMC4570173 26171799

[34] Rask E, Walker BR, Söderberg S, Livingstone DE, Eliasson M, Johnson O, et al. Tissue-specific changes in peripheral cortisol metabolism in obese women: increased adipose 11beta- hydroxysteroid dehydrogenase type 1 activity. J Clin Endocrinol Metab. 2002;87(7):3330-6. 10.1210/jcem.87.7.8661 12107245

[35] Tsilchorozidou T, Honour JW, Conway GS. Altered cortisol metabolism in polycystic ovary syndrome: insulin enhances 5alpha-reduction but not the elevated adrenal steroid production rates. J Clin Endocrinol Metab. 2003;88(12):5907-13. 10.1210/jc.2003-030240 14671189

[36] Neslihan SA, Betül EB, Bülent B, Sonat DK. Relationship between TSH and cortisol levels in euthyroid obese subjects. Endocrine Abstracts. 2015;37 EP623.

[37] Rezzónico J, Rezzónico M, Pusiol E, Pitoia F, Niepomniszcze H. High prevalence of thyroid nodules in patients with acrochordons (skin tags). Possible role of insulin-resistance. Medicina (B Aires). 2009;69(3):302-4. 19622476

[38] Farishta F, Farishta S. Insulin resistance and thyroid hypofunction in obese women - A cross sectional study. Integr Obes Diabetes. 2015;1(4):101-2.

[39] Vanderpump MPJ. The epidemiology of thyroid disease. British Medical Bulletin. 2011;99(1):39-51. 10.1093/bmb/ldr030 21893493

[40] Rezzonico J, Rezzonico M, Pusiol E, Pitoia F, Niepomniszcze H. Introducing the thyroid gland as another victim of the insulin resistance syndrome. Thyroid. 2008;18(4):461-4. 10.1089/thy.2007.0223 18346005

[41] Hegedüs L, Bonnema SJ, Bennedbaek FN. Management of simple nodular goiter: Current status and future perspectives. Endocr Rev. 2003;24(1):102-32. 10.1210/er.2002-0016 12588812

